# The Effects of Particle LET and Fluence on the Complexity and Frequency of Clustered DNA Damage

**DOI:** 10.3390/dna4010002

**Published:** 2024-01-05

**Authors:** Mohammad Rezaee, Amitava Adhikary

**Affiliations:** 1 Department of Radiation Oncology and Molecular Radiation Sciences, School of Medicine, Johns Hopkins University, 1550 Orleans St., Baltimore, MD 21231, USA; 2 Department of Chemistry, Oakland University, 146 Library Drive, Rochester, MI 48309, USA

**Keywords:** clustered damage, DNA-radical, LET, fluence, radiation, low energy electron, RBE, strand breaks, Monte-Carlo calculations

## Abstract

**Motivation:**

Clustered DNA-lesions are predominantly induced by ionizing radiation, particularly by high-LET particles, and considered as lethal damage. Quantification of this specific type of damage as a function of radiation parameters such as LET, dose rate, dose, and particle type can be informative for the prediction of biological outcome in radiobiological studies. This study investigated the induction and complexity of clustered DNA damage for three different types of particles at an LET range of 0.5–250 keV/μm.

**Methods:**

Nanometric volumes (36.0 nm^3^) of 15 base-pair DNA with its hydration shell was modeled. Electron, proton, and alpha particles at various energies were simulated to irradiate the nanometric volumes. The number of ionization events, low-energy electron spectra, and chemical yields for the formation of °OH, H°, ${\text{e}}_{\text{aq}}^{-}$, and H_2_O_2_ were calculated for each particle as a function of LET. Single- and double-strand breaks (SSB and DSB), base release, and clustered DNA-lesions were computed from the Monte-Carlo based quantification of the reactive species and measured yields of the species responsible for the DNA lesion formation.

**Results:**

The total amount of DNA damage depends on particle type and LET. The number of ionization events underestimates the quantity of DNA damage at LETs higher than 10 keV/μm. Minimum LETs of 9.4 and 11.5 keV/μm are required to induce clustered damage by a single track of proton and alpha particles, respectively. For a given radiation dose, an increase in LET reduces the number of particle tracks, leading to more complex clustered DNA damage, but a smaller number of separated clustered damage sites.

**Conclusions:**

The dependency of the number and the complexity of clustered DNA damage on LET and fluence suggests that the quantification of this damage can be a useful method for the estimation of the biological effectiveness of radiation. These results also suggest that medium-LET particles are more appropriate for the treatment of bulk targets, whereas high-LET particles can be more effective for small targets.

## 1. Introduction

The biological impact of ionizing radiation on living organisms causes detrimental outcomes such as lethality, carcinogenesis, and mutagenesis [1–6]. The prediction of the biological outcome has always been an imperative need for a wide range of medical and industrial applications, such as radiation therapy for cancer patients and protection from radiation sources in medicine, nuclear reactors, and space travel. Relative Biological Effectiveness (RBE) is an empirical parameter, which has widely been used in radiobiological studies to approximate biological outcomes from different types and qualities of radiation [1]. The use of RBE in practical applications such as clinical practice is, however, limited due to the uncertainty in measuring the RBE value, which results from its dependency on multiple parameters in addition to radiation dose, such as LET, fluence, and fluence rates [1]. With nuclear DNA being known as the principal cellular target of radiation, numerous studies have reported that physical and chemical changes in DNA resulting from irradiation can have substantial effects on cellular function, thereby nuclear DNA damage has been correlated with the biological effectiveness of radiation [2–4]. As an alternative to RBE measurement, the characterization of early biological events, i.e., radiation-induced DNA damage and its repair processes, have also been investigated to estimate biological responses to radiation [5].

Radiation-induced DNA damage is categorized into isolated and complex lesions with respect to their biological impact. Isolated damage such as single-strand break (SSB) and base lesions are known as sublethal damage that can be repaired efficiently by cellular mechanisms [3–5]. Complex damage known as clustered damage is the formation of two or more lesions on opposing DNA strands within a critical length of DNA, which is suggested to be within 10 to 20 adjacent base-pairs (bp), equivalent to one or two turns of the DNA helix [6]. The proximity of these lesions makes them less accessible to repair enzymes and their repair processes are consequently impeded [7]. The significant extent of clustered damage is observed to be the non-DSB (double-strand break) lesions (i.e., other type of lesions but not DSB e.g., base damage, abasic (AP) sites, and SSBs) [8]. High-LET radiations such as alpha particles are suggested to increase the complexity of clustered damage by inducing a high number of lesions in the critical length of the DNA-helix [9,10]. We note that the track of a high-LET particle has a very short mean free path that produces high ionization density along its track [1,2,9]. These ionization events initiate the generation of low-energy electrons (LEEs), free radicals (cation, anion, and neutral), and other reactive species that may react with DNA and induce various types of DNA-lesions [11]. It is well established that clustered DNA-lesions are predominantly induced by ionizing radiation [12,13]. The quantification of this damage and its complexity, including the type and extent of the lesions, has been studied to estimate the biological effects of radiation, particularly for high-LET particles and ions [14]. The experimental measurement of the clustered lesions is, however, challenging due to the variety and proximity of lesions along with their distribution throughout the genome [10].

Computational methods, such as the Monte-Carlo (MC) track structure, have been developed to estimate clustered DNA damage by calculating spatial energy deposition in nanometric (nm) volumes and estimating ionization distribution [15]. These calculations are typically performed in a single medium, most commonly in water, due to the limited data for the interaction cross-sections in different materials and calculation time in complex media. To estimate the spatial distribution of energy deposition and ionization events in DNA, the track structure calculations are superimposed on DNA-geometry models that have been developed at high molecular and atomic resolution within the nucleosome and chromatin structures [16,17]. In these simulations, each ionization overlaid on DNA is assumed to induce an SSB and the proximity of ionizations is considered for the DSB formation. These assumptions overlook the variety of radiation-induced DNA-lesions and the role of the physico-chemical, chemical, and biochemical processes involved in DNA damage formation [18,19]. Nevertheless, these assumptions are presently required to simplify the radiation-induced DNA damage simulation in the absence of accurate and time-efficient models for the calculation of different DNA-lesions induced by each reactive species such as LEEs and free radicals [20,21].

Calculations based on track structure simulate elastic and inelastic interactions of primary and secondary particles with matter, event by event, until the energies of the particles reach, theoretically, to the thermalization energy. At present, these calculations involve particle transport to the particle’s sub-ionization energy (e.g., 17.5 eV for electrons [22]) due to the lack of an efficient model for low-energy particle transport in matter. These low-energy particles, especially LEEs, are generated copiously (on the order of 5 × 10^16^ LEEs per Gy (Gy (Gray) is defined as the absorbed energy per unit mass of the object; Gy is the SI unit of absorbed dose)) in biological matter and can induce a variety of DNA-lesions via the rupture of chemical bonds between DNA-subunits [23]. The track structure can calculate the production of LEEs and their initial energy spectrum resulting from ionization events in a well-defined medium [24]. However, it does not include further inelastic interaction of LEE with a molecule through resonant and non-resonant scattering, e.g., dissociative electron attachment (DEA) [25,26]. Physico-chemical and chemical models have also been developed for the MC track structure to calculate the production of water radiolysis species and their diffusion in water [27,28]. The free radical species, particularly the hydroxyl radical (°OH), hydrogen atom (H°), and hydrated electron (${\text{e}}_{\text{aq}}^{-}$), are highly reactive toward DNA and induce a variety of lesions such as base modifications, sugar damage leading to unaltered base release and strand breaks, alkali-labile sites, tandem lesions, inter- and intra-strand crosslinks, and DNA-protein crosslinks [19,29,30].

Although water represents a considerable fraction of cellular mass and the water radiolysis species are highly reactive, the DNA-helix in a cell nucleus is highly packed with histone proteins and a higher order chromatin structure. In this configuration, nuclear DNA has a closely-bound hydration shell with less than 25 water molecules per nucleotide [31]. This structure reduces the indirect effects of radiation by protecting DNA from diffusible radicals. In addition, °OH rapidly reacts with other biomolecules and cellular components at rates close to its diffusion-controlled rates, thereby only those °OH created in the immediate vicinity of DNA have a significant probability to induce damage [32]. Track structure simulations have modeled physico-chemical and chemical stages in water to calculate the production of water radiolysis species, their diffusion, and reaction with each other [27,28]. However, to develop a specific model for accurate calculation leading to the formation of different types of DNA damage, the polymeric nature of the biomolecules as well as the proximate concentration of the reactive species and their corresponding rates of reactions with DNA have not yet been implemented in the MC track structures.

In the absence of accurate and time-efficient models to calculate probabilities for the formation of different types and amounts of DNA-lesions induced by the chemical reactive species and LEEs, measured yields and cross-sections of the polymer for the formation of the DNA-lesions can be utilized to quantify the lesions as a function of radiation parameters. The work presented in this report employs a semi-empirical approach to investigate the complexity and frequency of clustered damage in an nm-volume of hydrated DNA as a function of LET for three different particles including alpha particles, proton, and electron. This approach utilizes MC track structure to calculate the number of ionization events and the generation of LEEs and chemical reactive species with their distribution in the hydrated DNA. Then, experimentally measured yields for the formation of various DNA-lesions are used to estimate DNA damage complexity and variety. Two parameters named cluster size and damage site per single voxel volume, due to a single track, are defined to characterize the impact of particle LET and fluence on the complexity, type (variety), and frequency of clustered damage.

## 2. Materials and Methods

### 2.1. Radiation Transport Calculation

MC simulation was performed to model physical and physicochemical stages for the passage of electron, proton and alpha particles at different energies and LETs through hydrated DNA using the Geant4DNA toolkit (V.11.01) [33]. This Geant4DNA toolkit has both physics and chemistry models to calculate the stochastic partial energy deposition for both low- and high-energy particles, energy spectrum of the LEEs, reactive species from water radiolysis, and chemical reactions between the species followed by the radiolysis in nm-volumes [34]. This study used the G4EmDNAPhysics_option2 and G4EmDNAChemistry_option3 for the radiation transport calculation [35–38]. For these experiments, radiation beam was defined in a parallel plane field at the surface of the voxel with a straight direction into the voxel. The width of the field was one third of the length of the voxel side and located at the central region of the voxel side. Each experiment had at least 1000 independent histories.

Depending on the stage of the cell cycle in a eukaryotic cell nucleus, DNA exists in a variety of organizational states. However, in compact chromosomes, bulk water is largely excluded from the vicinity of the DNA [11,18,19]. Moreover, the very high global concentration (65 to 220 mg/mL) of macromolecules (DNA, RNA, proteins etc.) in the cell nucleus makes the role of direct-type effects of radiation in cells of crucial importance [39,40]. Therefore, hydrated DNA was modeled as a biological medium in a high-resolution nm-grid. Each voxel is a cube and has a volume of 36.0 nm^3^ to approximate the volume of 15bp DNA with its hydration shell including 20 water molecules per nucleotide. This, takes into account of the first and second layers of hydration in DNA [11,18,19].

This nm-volume was assumed as a critical volume, in which two or more lesions have the proximity to form clustered DNA damage. Each nucleotide was composed of tetrahydrofuran (THF), thymidine-monophosphate (TMP), and either purine (PU) or pyrimidine (PY) molecules. Every voxel contains 30 THF, 30 TMP, 15 PU, 15 PY, and 600 H_2_O molecules. These compounds with H_2_O molecules are uniformly distributed in the voxel to build 15 bp hydrated DNA. The DNA density was adjusted to 1.4 g/cm^3^ [41,42].

The primary radiations of electron, proton, and alpha particles were simulated to irradiate the hydrated DNA. Ten different energies for each particle were considered to characterize the effect of LET on the formation of the reactive species and DNA damage. Table 1 shows the energies and LETs of the particles simulated in this study. The maximum energies of 500 keV for electrons and 100 MeV for protons were considered in this study as low-LET particles, representing higher energy electrons (6–15 MeV) and protons (200–250 MeV) that are commonly used for therapeutic applications. Since the voxel has nm dimension, LET distribution from a monoenergetic particle is not expected to change significantly in the voxel. However, the LET of the incident particle, referred to as initial LET, and track-averaged LET have been calculated and summarized in Table 1. Ionization and excitation cross-sections for the electron and proton impact on the DNA were utilized at energies >15 eV and >70 keV, respectively. For the alpha particle, the cross-sections for liquid water with the density scaled to 1.4 g/cm^3^ were used in the simulation since the cross-section data were not available for DNA.

Upon irradiation of the hydrated DNA at each particle energy, several parameters including the number of ionization events, energy spectrum of LEEs (<25 eV), and chemical yield of reactive species were scored in each nm-voxel. The number of ionization events in each voxel was used to estimate the quantity of DNA-radicals (i.e., cation radical). These ionization numbers were calculated based on the ratio of volumes between non-hydrated and hydrated DNA. This ratio was 0.5 with the assumption that the average volumes of hydrated DNA and each water molecule were 36 nm^3^ and 0.0299 nm^3^, respectively. LEEs and DNA cation radicals were considered to calculate the direct action of radiation on DNA damage formation. The generation and temporal distribution of initial water radiolysis reactive species including °OH, H°, and ${\text{e}}_{\text{aq}}^{-}$ as well as H_2_O_2_ were scored in each voxel in different time scales from picoseconds (ps) to microseconds (μs). These reactive species were considered to estimate the indirect action of the particles on DNA damage formation.

### 2.2. DNA Damage Calculation

Experimentally measured cross-sections and yields for the impact of *LEEs* and the chemical reactive species on the formation of DNA damage were utilized to quantify different types of DNA lesions including SSB, DSB, and base damage in the DNA voxels. These measured data were used in the absence of a realistic model for the calculation of DNA damage induced by LEEs and the chemical species. Mean cross-sections for sub-ionization LEEs (1–15 eV) impact on the DNA double helix leading to stand break and base lesions were considered as 3.8 × 10^−2^ and 4.8 × 10^−2^ per nm^2^, respectively [43,44]. Yields for *SSB* induction by DNA cation radicals and the water radiolysis reactive species (i.e., °OH, H°, and ${\text{e}}_{\text{aq}}^{-}$) were considered to be 2.1 × 10^−2^ and 1.2 10^−2^ per eV energy deposition, respectively [45–49]. The yield for the formation of base lesions by the radicals from water radiolysis was 1.36 × 10^−2^ per eV energy deposition [46]. By calculating the quantity of intermediate species in each voxel and the yield for the formation of each specific type of damage, the numbers of the different DNA-lesions were calculated.

The complexity of clustered damage in a cellular nucleus was also simulated as a function of track-averaged LET. A spherical nucleus with a 4.0 μm diameter was modeled to contain the nm voxels. The irradiation of the nucleus by each primary particle at the different energies was simulated to deposit a fixed energy of 260 keV, equivalent to 2.0 Gy, in the nucleus. The radiation field for nucleus irradiation was also a parallel plane located at the surface of the nucleus. The number of voxels with at least one lesion (i.e., damage site) and quantity of lesions in each damage site (i.e., cluster size) were scored to evaluate the complexity of clustered DNA damage and the number of independent damage sites.

### 2.3. Statistical Uncertainty

All simulations were run on an OSX system with an M1 Max processor including a total number of 10 cores. Statistical uncertainties for the MC simulations of ionization events, LEE spectra and quantity, yields of reactive species as a function of time, LET, and particle type were within 3%. The uncertainty in the measured yields of DNA-lesions were considered to be in the range of 3–20%, according to the published reports [43,44,50–53].

## 3. Results

### 3.1. Ionization Density

**Figure 1.**
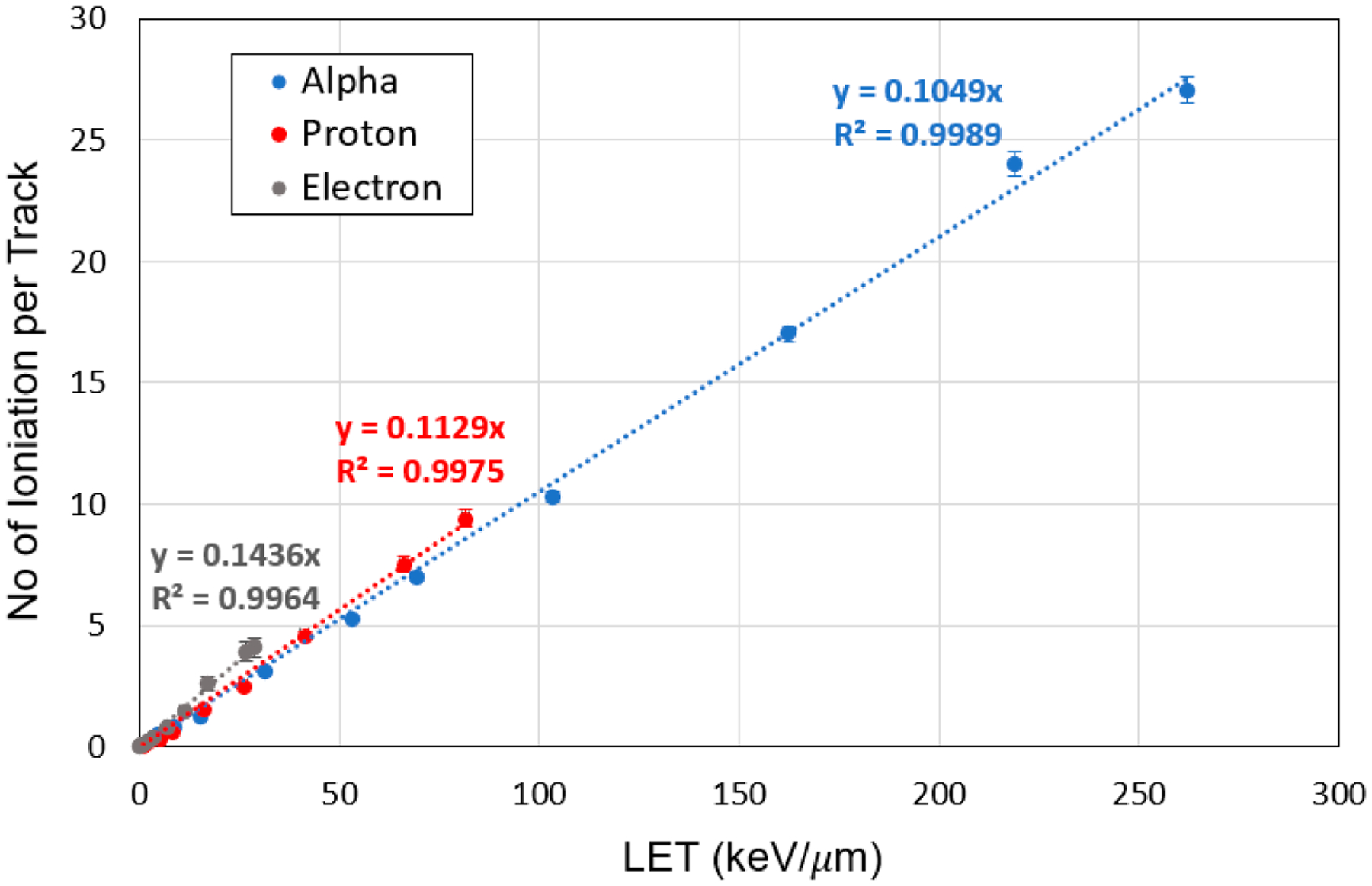
Number of ionizations as a function of track-averaged LET for a single track of electron, proton, and alpha particles passing through a critical volume of hydrated DNA (36.0 nm^3^) containing 15 base-pairs of DNA with water molecules.

The number of ionizations as a function of LET is calculated for various particle energies. In Figure 1, the ionization events due to the passage of a single track of alpha particles, protons, and electrons at different LETs through the nm-voxel containing the hydrated DNA is shown. Ionization number increases linearly with LET at the rates of 0.10, 0.11, and 0.14 per keV/μm for alpha particles, protons, and electrons respectively. The regression linear model fits well to the data with the coefficient of determination (R^2^) higher than 0.995 in all cases. In a single nm-voxel, one track of protons and electrons at their maximum LET can induce 9.4 and 4.4 ionizations, respectively. The number of ionizations for the alpha particle at the LETs of 162 keV/μm is calculated as 17.0 in the voxel. At a given LET, the number of ionization events is higher for electron than proton and alpha particles, especially at LETs larger than 8.5 keV/μm. Similarly, protons at LETs higher than 27.0 keV/μm generate more ionization events compared to alpha particles at a corresponding LET.

### 3.2. Characterization of LEEs

**Figure 2.**
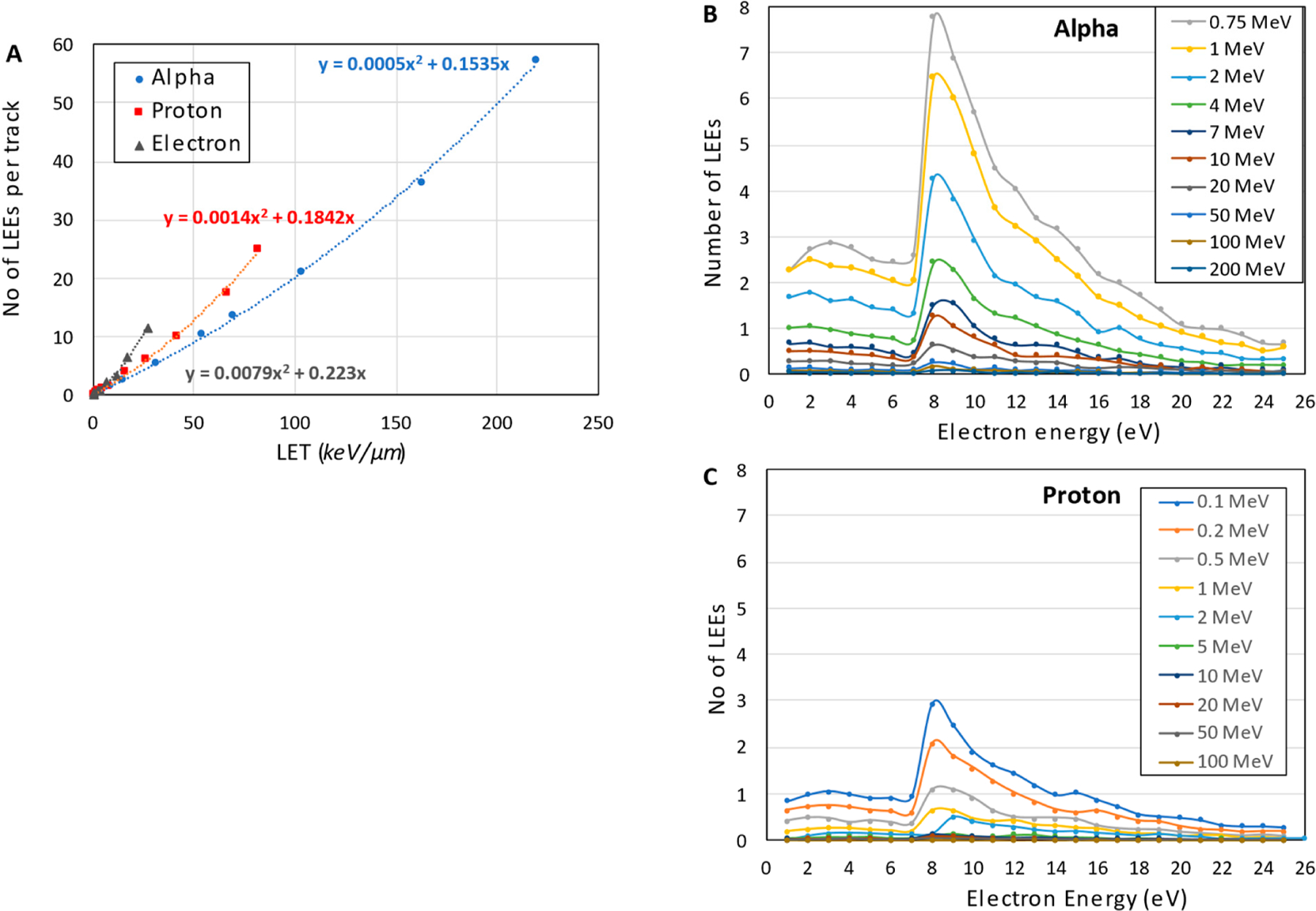
Generation of LEEs by a single track of electron, proton, and alpha particles at different LETs passing through the critical volume of DNA. (**A**) Total number of the generated LEEs as a function of LET and (**B**) the energy spectrumof LEEs generated by alpha and (**C**) proton particles at different energies.

The energy spectrum and quantity of the LEEs within the range of 1–25 eV are evaluated as a function of LET in the nm-voxel of hydrated DNA. Figure 2A shows the total number of LEEs generated by the passage of a single track of electron, proton, or alpha particles at different LETs through the hydrated DNA voxel. As expected, the number of LEEs increases with LET; however, this increase differs among the particle types.

At a given LET, electron and alpha particles produce the most and the least quantity of LEEs, respectively. Figure 2A also shows that the increase in the number of LEEs has a quadratic relationship with LET for all three particles.

Figure 2B,C show the energy spectra of the LEEs generated by the passage of a single track of proton and alpha particles at different energies through the nm-voxel. These spectra have similar shapes including a shallow and broad peak between 2–6 eV, a large narrow peak at the energies of 8–11 eV, and a rapid decrease in the number of LEEs beyond 15 eV. The number of LEEs reduces at higher energies of the incident particles because of the smaller LETs of these high-energy particles, resulting in the lower number of ionizations in the voxel. Our calculations predict that alpha particles generate a higher number of LEEs compared to protons due to its larger LET that is in agreement with experiment [54]. Figure 2B,C also show that the shape of LEE spectra is independent of the type, energy, and LET of the incident particles.

### 3.3. Reactive Species from Water Radiolysis

**Figure 3.**
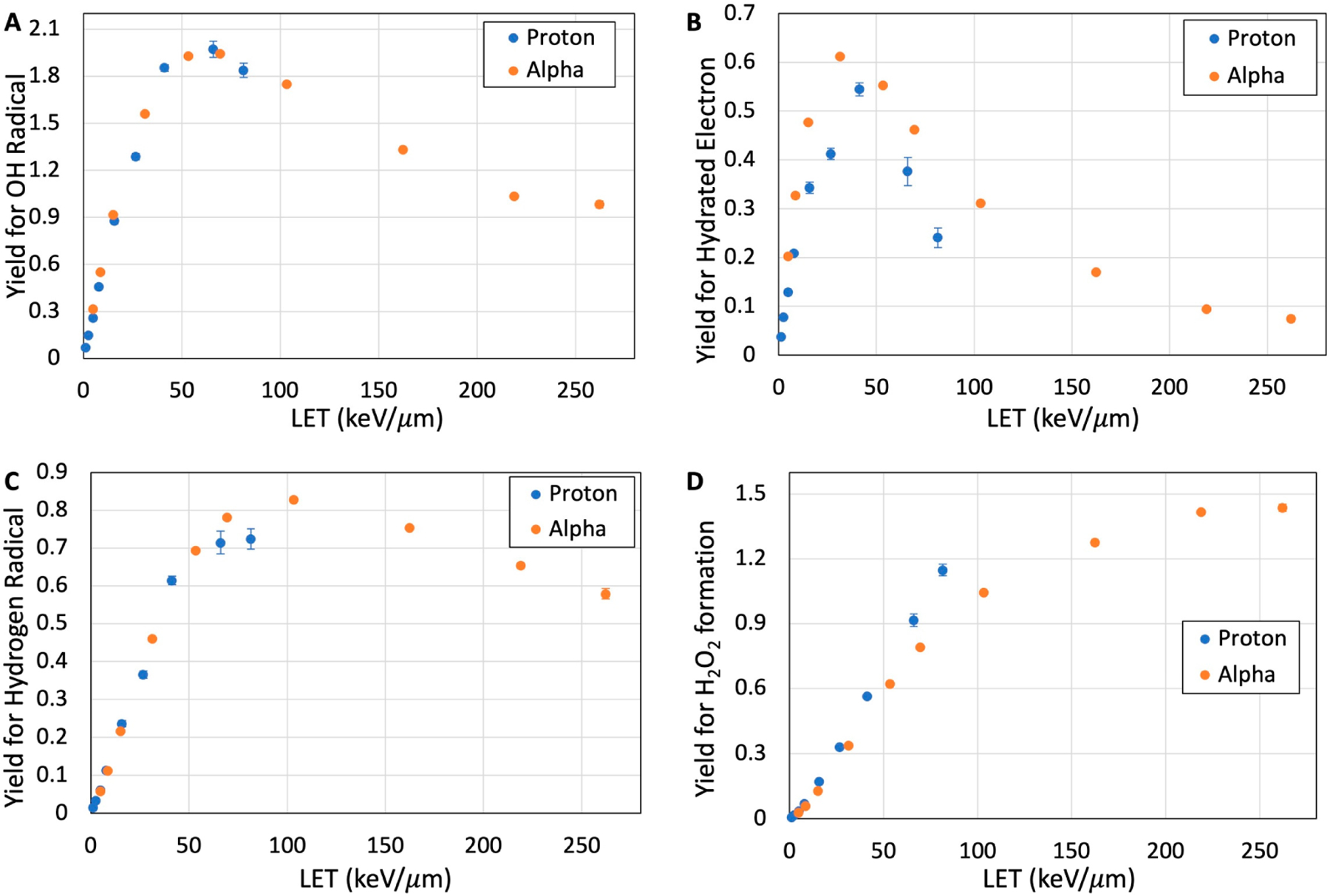
Yield (G-value) as a function of LET for the formation of ◦OH (**A**), ${\text{e}}_{\text{aq}}^{-}$ (**B**), H◦ (**C**), and H_2_O_2_ (**D**) from 100 eV energy deposition in the nanometric voxels by proton and alpha particles at 100 ns.

Yields for the formation of several reactive species including °OH, H°, ${\text{e}}_{\text{aq}}^{-}$, and H_2_O_2_ are calculated at different time scales and LETs in the nm-voxel. Based on time-dependent radical formation, G-values remain almost constant after 10 nanoseconds (ns). Figure 3A–D indicate the yield of formation of these reactive species as a function of particle LET in the nm-voxel of liquid water at 100 ns after irradiation. At this time scale, the G-values of the reactive species are independent of time (refer to Supplementary Materials); this allows us to evaluate the effect of LET on the formation of the species. Our calculations point out that the yields of °OH rapidly increase with LET, up to 66 keV/μm, then decrease slowly at higher LETs; similar trends were observed in experiments [55]. The rapid increase in the °OH yields with LET are at the rates of 0.047 and 0.054 per keV/μm for proton and alpha particles, respectively. Similarly, the yields for the formation of H° and ${\text{e}}_{\text{aq}}^{-}$ (Figure 3B,C) show a rapid increase at lower LETs followed by a slight decrease at higher LETs. These results also represent that the LET required to produce the maximum yields of these reactive species is different. Among these reactive species, the maximal yield for ${\text{e}}_{\text{aq}}^{-}$ is at LETs as low as 30 keV/μm range, whereas the LET formaximal H° yield is as high as 100 keV/μm.

As expected, the yield for H_2_O_2_ formation in the nm-voxel increases with LET since H_2_O_2_ is a secondary species, primarily produced via the recombination of °OH [25,30]. Figure 3D shows that H_2_O_2_ yield increases linearly with LET, up to 100 keV/μm, then its increase continues at a lower rate. These results indicate that the probability of H_2_O_2_ formation due to intra-track recombination is negligible (<4%) at LETs lower than 5 keV/μm.

At a given LET, protons produce a higher yield of H_2_O_2_ compared to that of alpha particles. The maximum G-values of H_2_O_2_ formation are calculated as 1.15 and 1.44 for proton and alpha particles at their maximum LET, respectively, in the nm-voxel. Electrons expectedly produced the least amount of H_2_O_2_ due to their low LET relative to proton and alpha particles, with yields under 0.3 molecules per 100 eV. Yields for the formation of °OH, H°, and ${\text{e}}_{\text{aq}}^{-}$ in the nm-volume decrease with time due to subsequent reactions after their production. As opposed to these species, the yield for the formation of H_2_O_2_ increases with time and reaches a maximum level at times longer than 10 ns. The results of these yields as a function of time for each particle LET are presented in the Supplementary Materials.

### 3.4. Quantification of DNA Damage

**Figure 4.**
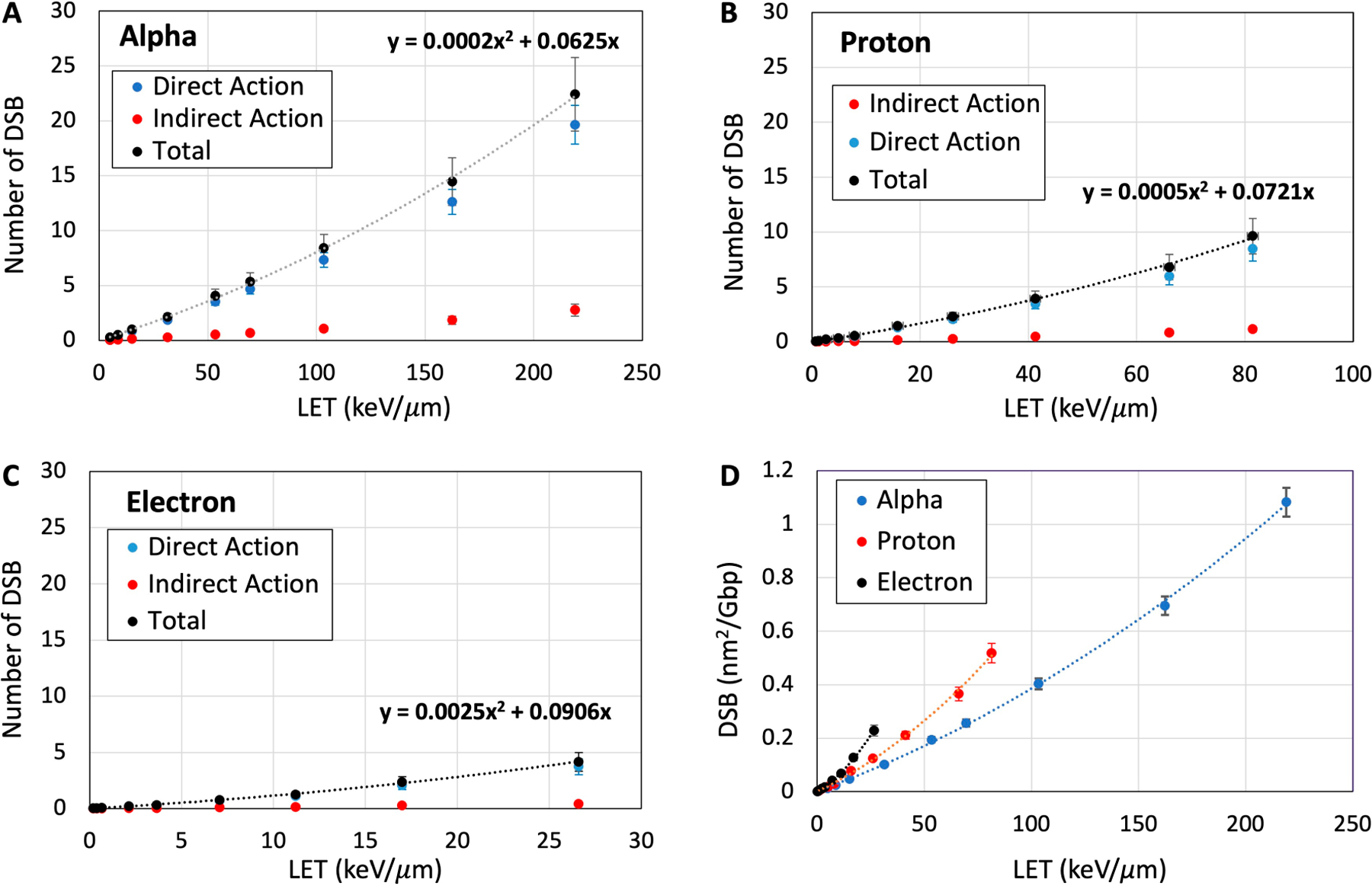
Number of DSBs as a function of LET induced by a single track of alpha particles (**A**), protons (**B**), and electrons (**C**) at different LETs passing through the critical nm-volume of DNA. Contribution of direct and indirect actions of the particles in the induction of DSBs is compared to the total number of DSBs. (**D**) Number of DSB per unit fluence and Gbp as a function of LET for alpha particles, protons, and electrons.

In this work, we have calculated different types of DNA-lesions including SSB, DSB, base damage, and clustered lesions in the DNA-voxels (see Section 2.2). DSBs and the total number of DNA-lesions are quantified in the nm-voxel of hydrated DNA. Figure 4A–C show the number of DSBs from the direct and indirect actions of the electron, proton, and alpha particles as a function of LET and compares them with the total number of DSBs. Both direct and indirect actions of the particles for the induction of DSBs increase with particle LET but at different rates. The rates of direct action with LET are higher by about an order of magnitude than those of indirect action. These theoretical predictions support the proposition of significant involvement of direct effect in radiation damage to cellular DNA that were obtained from electron paramagnetic studies and product analyses of irradiated hydrated DNA [11,19,50,51]. The total number of DSBs was mainly formed from the direct action of the particles at high LETs. The minimum track-averaged LETs of 9.0, 11.8, and 15.2 keV/μm are required to induce one DSB in a single voxel (volume = 36 nm^3^) by a single track of electron, proton, and alpha particles, respectively. This result suggests that electrons and protons are more efficient than alpha particles in the induction of DSBs at a given LET. Figure 4A–C also show that the total number of DSBs has a quadratic relationship with LET for each of the three particles.

Figure 4D shows the number of DSBs per giga base-pair with respect to the fluence and LET for electron, proton, and alpha particles. Since dose is a deterministic parameter and cannot be used for nm-voxel, the results of this figure can be used for comparison of DSB yield with those obtained from other reports. For example, a recent report on the microdosimetry of alpha particles using TOPAS-nBio shows a DSB yield [56] that is comparable to this study.

**Figure 5.**
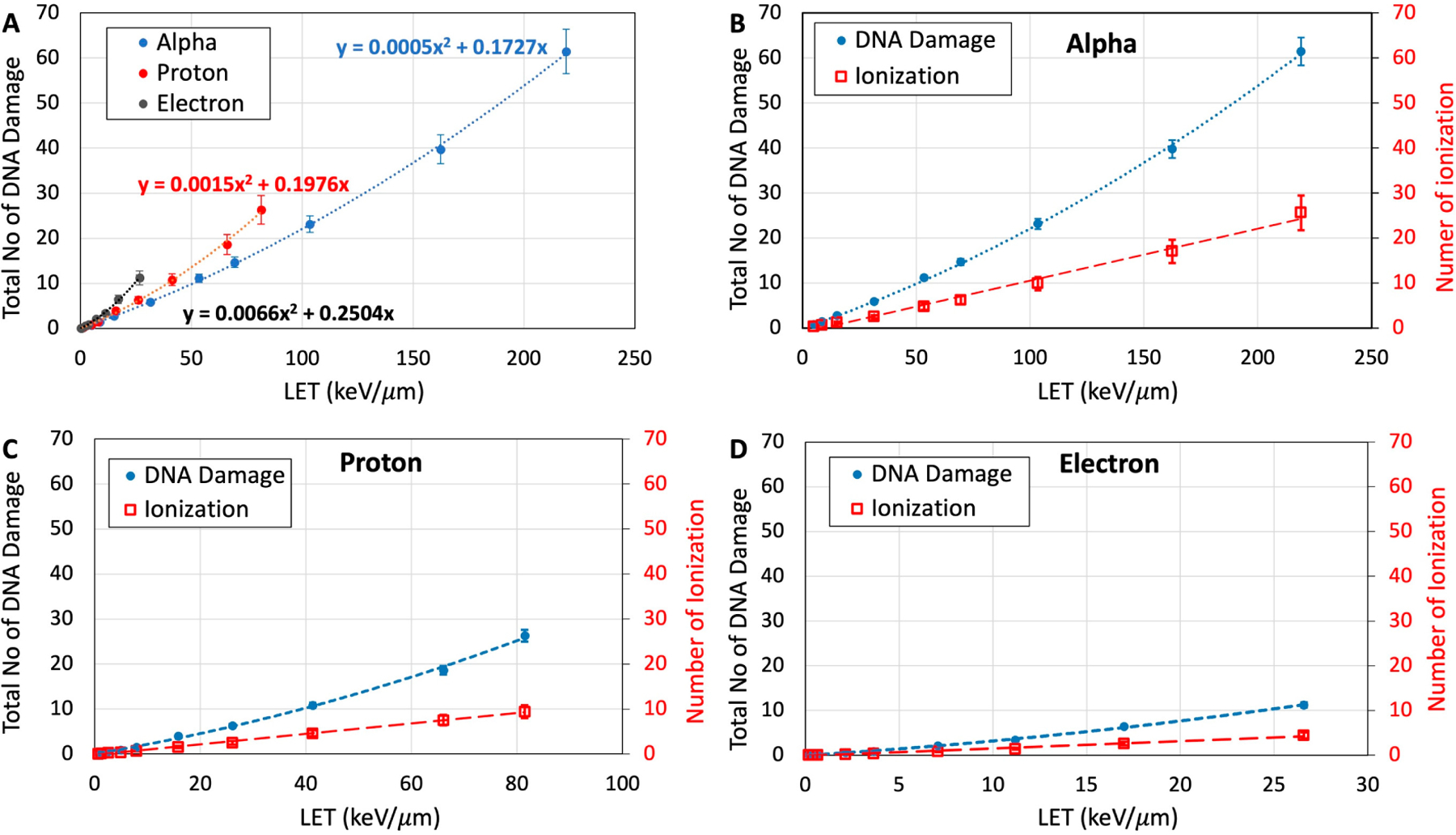
(**A**) Total amount of DNA damage including SSB, DSB, and base damage as a function of LET for a single track of electron, proton, and alpha particles passing through the nanometric volume of hydrated DNA. Comparison between total amount of DNA damage and total number of ionizations for a single track of alpha particles (**B**), protons (**C**), and electrons (**D**) in the nanometric volume of hydrated DNA.

The total number of DNA damage was quantified by the summation of all base lesions and strand breaks in the nm-voxel. Figure 5A shows total DNA damage resulting from the passage of a single track of the particles at different LETs through the hydrated DNA-voxel (volume = 36 nm^3^). Similar to DSB formation, the total number of DNA-lesions induced by electrons is calculated to be higher than those induced by proton and alpha particles at a given LET. Protons also induce a higher number of DNA-lesions compared to alpha particles, particularly at LETs higher than 20 keV/μm. The minimum LET required to induce a clustered damage containing at least two lesions due to a single track of electrons, protons, and alpha particles are 7.1, 9.4, and 11.5 keV/μm, respectively.

Figure 5B–D compare the total DNA damage with the total number of ionization events for each of the particles. These results show that both DNA damage and ionization events increase with LET but at different rates. Increases in DNA damage can be fitted with a quadratic function whereas ionizations have linear fits. This finding corroborates the experimental observations on the DNA-lesions observed in 193 nm laser irradiated plasmid DNA (pTZ18R) at higher intensity [57]. This linear-quadratic nature of the DNA damage could occur by two-event induction (e.g., cation radical coupled with excited states and excited anion radical in close proximity) along with, e.g., the radical swing-over process (similar to the Siddiqui-Bothe mechanism that was originally proposed for indirect effect) via one-event induction [19,25,47,48]. Also, a pulsed electron-electron double resonance (PELDOR) spectroscopic study of ion-beam irradiated DNA showed that there was an average of 17.7 ± 0.7 radicals per spherical cluster with a cluster radius of 6.79 nm in agreement with the track structure calculations [11,19,58].

### 3.5. Cluster Size and Damage Site

Cluster size represents the number of lesions in a single nm-voxel (volume of each voxel = 36 nm^3^ (see Section 2)), and its value depends on the particle LET. Damage site describes the quantity of the nm-voxels with at least one DNA-lesion, and it predominantly depends on the number of particle tracks (i.e., fluence).

**Figure 6.**
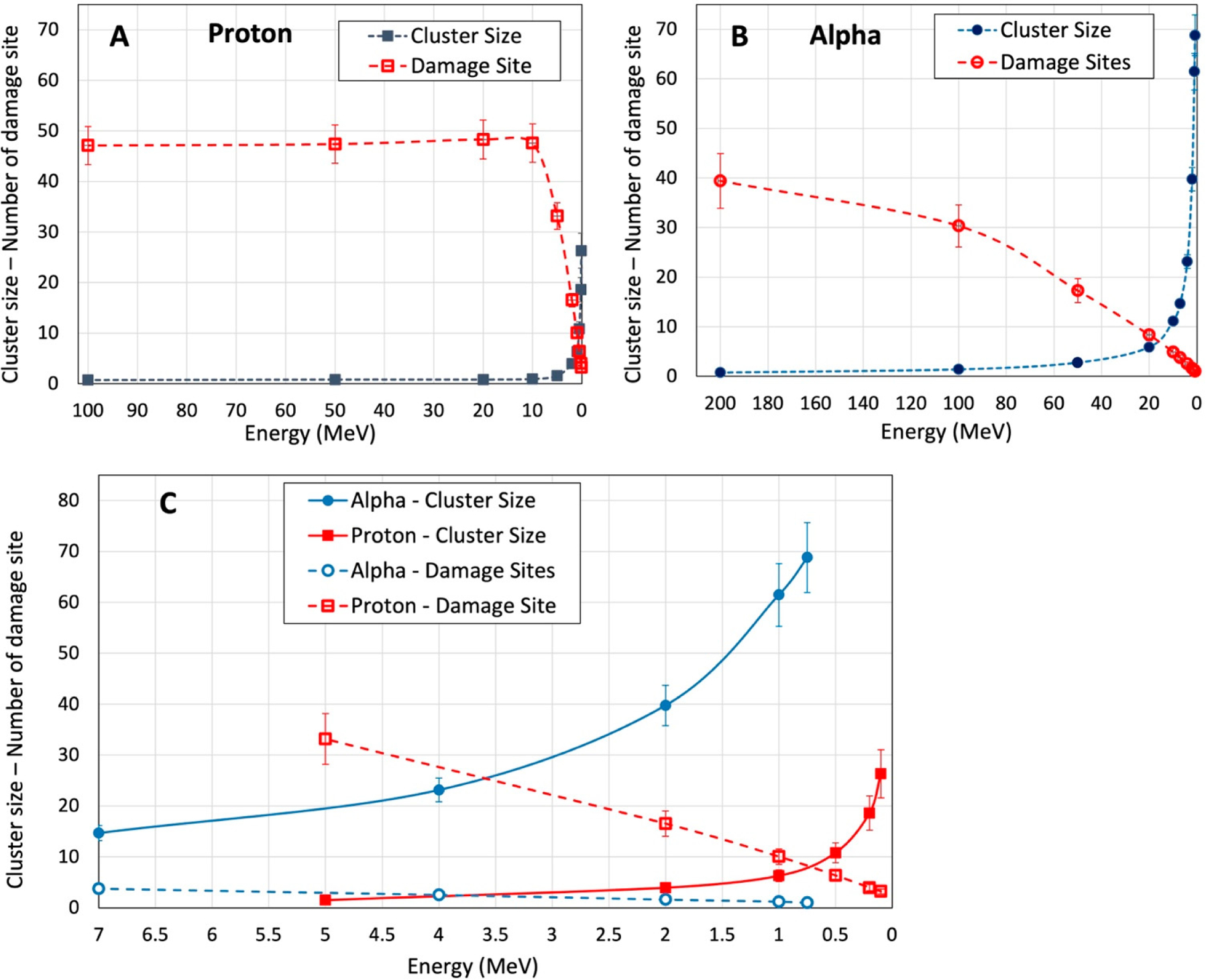
Cluster size and damage site as a function of the incident energy of proton (**A**) and alpha particles (**B**). DNA damage induced by the 260 keV energy deposition (i.e., 2 Gy) of these particles in a 4-μm diameter nucleus. (**C**) Cluster size and damage site induced by proton and alpha particles at incident energies less than 7 MeV. Here the number of cluster size is per voxel volume (i.e., 36 nm^3^) and the damage sites are per 4 μm diameter. The volume of a voxel was assumed as a critical volume, in which two or more proximate lesions form a clustered DNA damage (see Section 2).

These two parameters are defined to characterize the impact of LET and fluence of incident particles on the complexity of DNA lesions and distribution of the nm-voxels containing DNA damage. Figure 6A–C show the cluster size and number of damage sites from a 260 keV energy deposition in a cellular nucleus from the alpha particles, protons, and electrons at different energies assuming the particle tracks pass through chromosomes and DNA molecules. This energy deposition is equivalent to the therapeutically relevant 2.0 Gy absorbed dose in a 4 μm diameter nucleus. Electrons are not included in Figure 6 because the energies required for electrons passing through the nucleus are higher than 30 keV. At these energies, electrons have low LETs (<1 keV/μm).

At 10 MeV and higher energies that are equivalent to LETs smaller than 5 keV/μm, protons have a similar number of damage sites to the concomitant cluster size of one or lower (Figure 6A). These results also suggest that the largest number of DNA-lesions induced by the high-energy protons should lead to one damage site in the critical nm-volume of hydrated DNA. This agrees with other studies on low-LET radiations [49–53,56]. With the decrease in the proton energy under 10 MeV (LET > 5 keV/μm), the cluster size increases exponentially, while the damage sites decrease linearly. Figure 6B shows similar behaviors for cluster size and damage sites induced by alpha particles. At high energies (>150 MeV) where LET is smaller than 7 keV/μm, alpha particles induce a large number of damage sites with minimum cluster size (<1.5). Cluster size increases exponentially at energies less than 100 MeV (>9 keV/μm) and the number of damage sites decreases linearly from 30 to 1. It should be noted that the inter-track recombination of reactive species is negligible in this experimental setup. At the lowest LET radiation (i.e., 0.2 keV/μm for 500 keV electrons), the number of tracks passing through the nucleus is about 320 to deliver the 2 Gy dose. Regarding the volume of the nucleus and LET of the tracks, the nominal distance between two inelastic interactions from two adjacent tracks is about 0.1 μm. At this distance, the probability of inter-track recombination is very low. This finding supports the basis to explain the steady state radiolysis (e.g., gamma radiolysis) results using the results obtained employing pulse radiolysis provided the dose/pulse is not too high [30,59,60]. Moreover, this finding establishes that the yields of radiation-produced radicals are independent of dose rates and dose levels which were employed recently in ultrahigh dose-rate (FLASH) radiotherapy studies such as those reported by Favaudon et al. [61].

Figure 6C compares cluster size and damage sites for proton and alpha particles at energies less than 7 MeV. These particle energies represent the descending end of Bragg peak in the depth dose distribution of proton and radiopharmaceutical therapy using alpha particle-emitting radionuclides [62,63]. This comparison along with the results shown in Figure 5 show that alpha particles induce a lower number of damage sites with a larger number of DNA-lesions in each site, whereas protons produce more damage sites containing less DNA-lesions. Within the energy range of 10 MeV to <1 MeV, cluster size in the nm voxel increases to 26 and 69 for proton and alpha particles, respectively. In this energy range, the number of damage sites decreases from 33 to 3 for proton and from 5 to 1 for alpha particles. This trend has been observed experimentally in irradiated skin fibroblast cells [64]. In this work, the authors reported lower number of radiation-produced clusters or radiation-induced foci (RIF per Gy) for low-LET (e.g., X-rays) and very high LET particles (i.e., 600 MeV/n ^56^Fe).

## 4. Discussion

The biological effects of high-LET radiations have received much attention since last decade due to substantial growth in their therapeutic applications for external beam particle therapy and radiopharmaceutical therapy [65,66]. In these applications, the primary metric used in treatment planning systems for the prediction of treatment response is absorbed dose, similar to the therapeutic applications of low-LET radiations (e.g., megavoltage X-rays). Absorbed dose is derived from the deposition of ionization energy under specific conditions [67,68] and it does not include the spatial distribution of the ionization and reactive species generated by the ionization events. Thus, this metric does not describe the effects of radiation quality such as LET and dose rate. At present, clinical practices have adapted overly simplified qualifying factors such as dose modifying factor or RBE dose to approximate treatment outcome by scaling delivered dose. The proton treatment planning system, for example, considers a dose modifying factor of 1.1 for the tissue volumes located at the Bragg Peak of proton beam. The characterization of LET effects on biological systems using experimental measurement contains significant uncertainties and cannot be quantified reliably for practical applications [1]. The uncertainties partially arise from the sample dimension and continuous changes in the energy and LET of the particle track within the sample due to consecutive inelastic interactions. Therefore, results on biological outcome employ a range of particle LETs in which an average LET may not accurately represent the LET distribution in the irradiated sample and are used for the characterization of LET effects [69]. Selecting biological targets at the molecular level can alleviate this issue due to the small dimension of the target. In addition, the nm-voxel of hydrated DNA as the target provide a novel method for including high scavenging capacity of cellular environment on the radiation-mediated water-derived radicals. Current simulation methods limit diffusion of the water radicals to mimic the a few ns lifetimes of the radicals during the early track expansion for low LET radiation [33,38].

It is well known that high-LET particles induce complex molecular damage, e.g., clustered DNA-lesions, which may have a different biological response compared to simple damage induced by low-LET radiations [8,64,70,71]. The characterization of the damage as a function of LET can then be the initial step in their biological quantification due to high-LET radiations.

This study has introduced two parameters of cluster size and number of damage sites to better elucidate the relationship of clustered DNA damage with LET. Our results in Figure 6 suggest that each track of proton at LETs up to 9 keV/μm, i.e., equivalent to energies higher than 4.0 MeV, has a very low probability of inducing clustered damage (i.e., cluster size of two or more). With respect to the depth dose distribution of clinical beams, protons reach this energy at the Bragg peak. At a higher proton LET, which is produced at the end of the Bragg peak, clustered damage has a high probability of formation and its complexity increases exponentially with LET.

Larger cluster size (i.e., more complex damage) expectedly enhances the probability of cell death; however, the delivery of a high-LET particle beam to a target tissue such as a macroscopic volume of solid tumor raises two important radiobiological concerns. First, each high-LET track delivers a large amount of dose per track, resulting in a reduction in the number of tracks compared to the number of low-LET tracks for delivering a given dose. This means that a partial volume of the target does not directly interact with radiation. Secondly, the range of the high-LET tracks is very short and may pass through only a few cells. This makes very high-LET radiation such as alpha particles more appropriate for targeted delivery to microscopic volumes. With respect to the clustered DNA damage and its biological response, tissues located within a spread-out Bragg peak may have a complex response to the radiation because both low- and high-LET protons interact with the tissue in this region. This complicates the characterization and prediction of the biological response in irradiated tissue. Table 2 shows examples of alpha particles and protons at different energies, ranges, and LETs for the comparison of cluster size and number of damage sites in the high- and low-energy regions.

The limited number of tracks and short range of high-LET particles can reduce the number of irradiated cells in a tissue, which may cause a lower biological effectiveness of radiation in tissue. This would suggest a tradeoff between fluence and LET to achieve the optimum biological effectiveness by irradiating the majority of target cells with particle LET sufficient to induce clustered damage. Furthermore, these findings provide additional evidence that neither dose nor LET alone are an appropriate descriptor for the RBE or biological effectiveness of radiation [15,16].

We note here that recent study on clustered damage formation in plasmid DNA (pUC19) solution irradiated by Fe ion-beam (LET = 200 keV/μm) reported 2–3 damage sites with cluster size of 2–4 induced by a single track of the ion passing through a single plasmid DNA molecule [13]. Our calculations support this observation by predicting the formation of more than one damage site containing cluster size of 2 or more due to impact of a single track of alpha particles on the hydrated DNA.

This study also calculated the number of ionization events as a function of LET in the medium of hydrated DNA at the nm resolution. Our results in Figures 1 and 5 indicate that an increase in total DNA damage with LET follows the ionization number only at LETs lower than 3 keV/μm for electrons and 10 keV/μm for alpha and proton particles. At higher LETs, relevant to the Bragg Peak of charged particles and ions, total DNA damage deviates substantially from ionization number depending on the LET and particle type. Therefore, the number of ionization events underestimates the total number of DNA lesions for high-LET particles, suggesting that the scaling of absorbed dose using a modifying factor cannot accurately estimate the total amount of DNA damage. This finding accentuates the development of new approaches to compute and quantify DNA damage due to high-LET radiations.

LEEs are the most abundant and important reactive species for the induction of DNA damage, particularly for high-LET particles [23,72]. The energy distribution of LEEs in hydrated DNA, shown in Figure 4B,C, is similar to those simulated in water by Pimblott and LaVerne using the differential dipole oscillator strength distribution of water and DNA for electron and heavy particles [24,73]. A pronounced peak at 8–11 eV suggests the most probable electron energy for the interaction with the DNA. At these energies, LEEs can induce both isolated and clustered DNA damage [74,75]. The number of LEEs expectedly increases with LET due to the higher number of ionization events regardless of the type of incident particle. This study considered measured cross-sections for the formation of stand breaks and base damage at 9–10 eV, the most probable electron energy, as the nominal values for all LEEs in our first approximation. Research for the measurement of partial cross-sections at each electron energy for each DNA-subunit have been conducted for pure DNA and protein-DNA complexes [76–78]. These cross-section values can be incorporated into the DNA damage simulation for more accurate analysis, and to provide insight on the contribution of a single LEE in the formation of clustered damage.

In addition to LEEs, chemical reactive species formed from water radiolysis induce different types of DNA-lesions [30]. The yields for the formation of the reactive species have been measured and calculated as a function of dose and LET, mainly in bulk water [74]. Our results show that the yields for the reactive species in the nm-volume differ from those in bulk water. Both theoretical and experimental studies have shown that °OH, H°, and ${\text{e}}_{\text{aq}}^{-}$ have the highest yield for low-LET radiation in bulk water and the yields reduce with an increase in LET [79,80]. In the nm-volumes containing a limited number of water molecules, as shown in Figure 3, the yields at 100 ns increase with LET to a maximum value, then decrease at very high LETs. Increases in LET to very high values shorten the mean free path of the particle track, which reduces the distance between the radicals and reactive species, thereby enhancing the probability of their recombination. Consequently, the probability of the radiation-produced radicals that can diffuse and thereby leave the nm voxel gets reduced. Absolute values of the yields are time-dependent because of the diffusion and reaction rates (see Supplementary Materials) [81,82]. When the probability of the radical recombination (e.g., °OH) increases, the yield of the corresponding diamagnetic product (e.g., H_2_O_2_) also becomes higher. This increase in the yield with LET agrees with those observed in the measurement [83,84]. Work is underway to extend this model to take into account the experimental conditions (e.g., see ref. [45,48,49,85]) for the prediction of stable DNA-radical yields and their correlations with the diamagnetic products [11,19,45,47–51] in the chromatin structure [86]. Thus, this Radiation Track Structure Model would enable us to understand how the initial stochastic physical, physicochemical, and chemical events of radiation impact on biomolecules lead to damage that are determined experimentally. Moreover, as evident from the results presented in this work, our approach of combining modeling and experiments have important implications towards elucidating the mechanisms involved in new radiotherapy techniques such as FLASH radiotherapy and high-LET Particle Radiotherapy.

## 5. Conclusions

This study characterizes particle LET and fluence within nano-volume and their influence on the radical yields. These results affect the type, size, and extent of the clustered DNA damage. Furthermore, for the first time, this work investigates the simulation of LEE and the prediction of different types of DNA damage induced by LEE within the nano-volume due to a single track. With consideration of the stochastic nature of physicochemical and chemical processes involved in the interaction of radiation with DNA, our finding suggests that the quantity and spatial distribution of ionization events underestimate the number and complexity of DNA-lesions induced by high-LET particles. In addition, our work emphasizes that the interplay between LET and fluence should be considered in radiobiological studies. Consequently, the RBE of very high LET radiations can reduce due to the low fluence at a fixed dose delivered to a large target. As a result, medium-LET particles should be more appropriate for the treatment of bulk tumors, whereas high-LET particles can be more effective for small tumors.

## Supplementary Material

Supplementary material Figure S1: Yields for the formation of hydroxyl radicals as a function of time in the range of 1 ps to 1 μs for different proton LETs in a nm-volume (36.0 nm^3^), Figure S2: Yields for the formation of hydrated electrons as a function of time in the range of 1 ps to 1 μs for different proton LETs in a nm-volume (36.0 nm^3^), Figure S3: Yields for the formation of hydrogen atom as a function of time in the range of 1 ps to 1 μs for different proton LETs in a nm-volume (36.0 nm^3^), Figure S4: Yields for the formation of hydrogen peroxide as a function of time in the range of 1 ps to 1 μs for different proton LETs in a nm-volume (36.0 nm^3^), Figure S5: Yields for the formation of hydroxyl radicals as a function of time in the range of 1 ps to 1 μs by alpha particle at different LETs in a nm-volume (36.0 nm^3^), Figure S6: Yields for the formation of hydrated electrons as a function of time in the range of 1 ps to 1 μs by alpha particle at different LETs in a nm-volume (36.0 nm^3^), Figure S7: Yields for the formation of hydrogen radical as a function of time in the range of 1 ps to 1 μs by alpha particle at different LETs in a nm-volume (36.0 nm^3^), Figure S8: Yields for the formation of hydrogen peroxide as a function of time in the range of 1 ps to 1 μs by alpha particle at different LETs in a nm-volume (36.0 nm^3^).

## Figures and Tables

**Table 1.** Energy and LET of particles used in this study. Initial LET is the LET of the incident particle at the entrance of each voxel and track-averaged LET is calculated within each voxel.

Alpha			Proton			Electron		
Energy (MeV)	Initial LET (keV/μm)	Track-Averaged LET (keV/μm)	Energy (MeV)	Initial LET (keV/μm)	Track-Averaged LET (keV/μm)	Energy (keV)	Initial LET (keV/μm)	Track-Averaged LET (keV/μm)
0.75	233.8	262.2	0.1	70.6	81.5	0.1	28.7	28.7
1	219	221.7	0.2	66.0	66.2	0.2	26.6	28.5
2	161.4	162.4	0.5	41.0	41.3	0.5	17	18.6
4	101.3	103.4	1	26.7	28.0	1	11.2	12.5
7	67.4	69.5	2	15.9	16.9	2	7.1	7.3
10	51.5	53.4	5	7.9	8.8	5	3.6	3.8
20	30.6	31.4	10	4.8	5.0	10	2.1	2.2
50	14.6	15.2	20	2.6	2.8	50	0.7	0.7
100	8.1	8.6	50	1.2	1.3	100	0.4	0.4
200	4.5	4.9	100	0.7	0.7	500	0.2	0.2

**Table 2.** Cluster size and number of damage sites resulting from low- and high-energy electron, proton and alpha particles irradiating a 4 μm diameter nucleus. Cluster size is the number of lesions per voxel volume (i.e., 36 nm^3^, Section 2). The volume of a voxel was assumed as a critical volume, in which two or more proximate lesions form a clustered DNA damage (see Section 2). The damage sites are per 4 μm diameter. The nucleus received 2.0 Gy dose from each of the particles considered in this work.

Particles	Low Energy, High LET Particles					High Energy, Medium- and Low-LET Particles				
	Energy (MeV)	LET (keV/μm)	Range (keV/μm)	Cluster Size per Voxel Volume	Number of Damage Site	Energy (MeV)	LET (keV/μm)	Range (keV/μm)	Cluster Size per Voxel Volume	Number of Damage Site
α	2	162.4	11.2	39.8	1.6	50	15.2	1.8	2.8	17.3
	4	103.4	27.1	23.1	2.5	100	8.6	6.4	1.4	30.3
p	0.5	41.3	8.9	10.8	6.3	50	1.2	22.3	<1	47.4
	1	26.1	24.6	6.3	10.1	100	0.7	77.2	<1	47.1
e	NA	NA	NA	NA	NA	0.1	0.4	0.14	<1	67.9
						0.5	0.2	1.8	<1	86.7

## Data Availability

The original data are available for share upon request to the authors by email.
