# Supplementary material for: The Effects of Particle LET and Fluence on the Complexity and Frequency of Clustered DNA Damage
Source: DNA (Basel). Author manuscript; Available in PMC 2024 Mar 1. (PMC10810015; doi:10.3390/dna4010002)
Supplement: Supplementary material — Figure S1: Yields for the formation of hydroxyl radicals as a function of time in the range of 1 ps to 1 μs for different proton LETs in a nm-volume (36.0 nm3), Figure S2: Yields for the formation of hydrated electrons as a function of time in the range of 1 ps to 1 μs for different proton LETs in a nm-volume (36.0 nm3), Figure S3: Yields for the formation of hydrogen atom as a function of time in the range of 1 ps to 1 μs for different proton LETs in a nm-volume (36.0 nm3), Figure S4: Yields for the formation of hydrogen peroxide as a function of time in the range of 1 ps to 1 μs for different proton LETs in a nm-volume (36.0 nm3), Figure S5: Yields for the formation of hydroxyl radicals as a function of time in the range of 1 ps to 1 μs by alpha particle at different LETs in a nm-volume (36.0 nm3), Figure S6: Yields for the formation of hydrated electrons as a function of time in the range of 1 ps to 1 μs by alpha particle at different LETs in a nm-volume (36.0 nm3), Figure S7: Yields for the formation of hydrogen radical as a function of time in the range of 1 ps to 1 μs by alpha particle at different LETs in a nm-volume (36.0 nm3), Figure S8: Yields for the formation of hydrogen peroxide as a function of time in the range of 1 ps to 1 μs by alpha particle at different LETs in a nm-volume (36.0 nm3). [file NIHMS1959035-supplement-Supplementary_material.zip › dna-2695584-supplementary.pdf]

Supplemental Materials

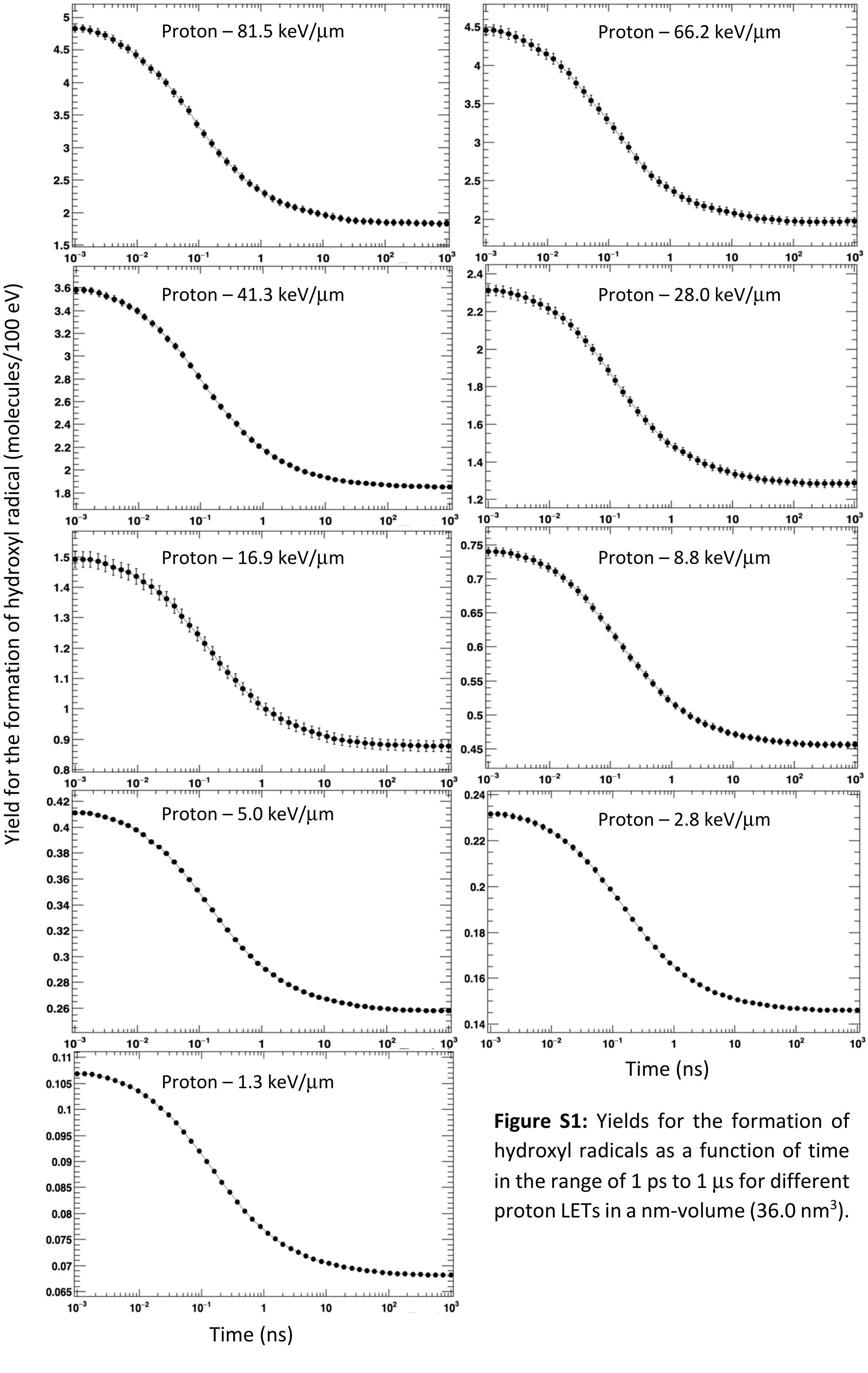

Figure S1: Yields for the formation of hydroxyl radicals as a function of time in the range of 1 ps to 1 μs for different proton LETs in a nm-volume (36.0 nm^3).

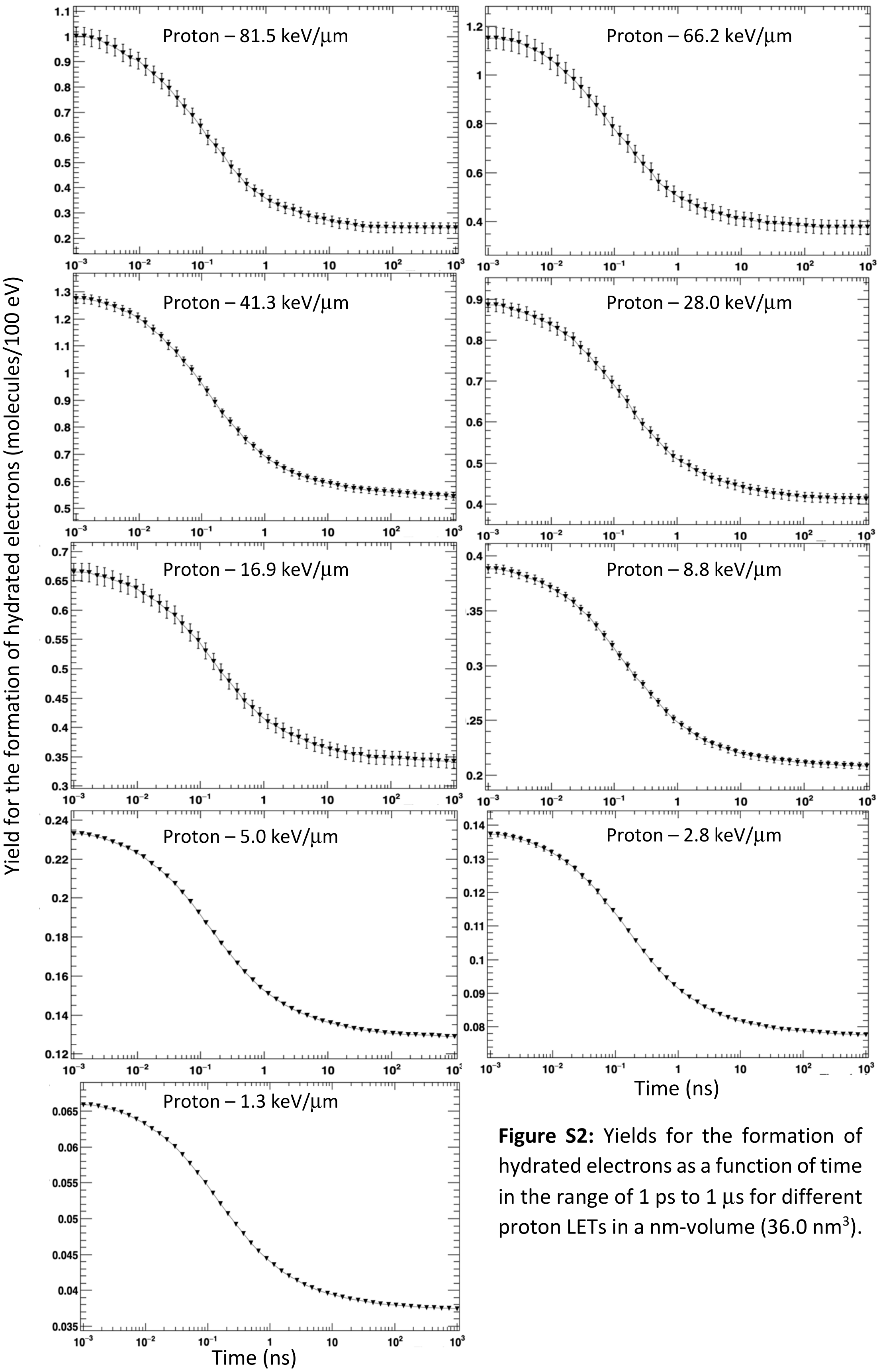

Figure S2: Yields for the formation of hydrated electrons as a function of time in the range of 1 ps to 1 μs for different proton LETs in a nm-volume (36.0 nm^3).

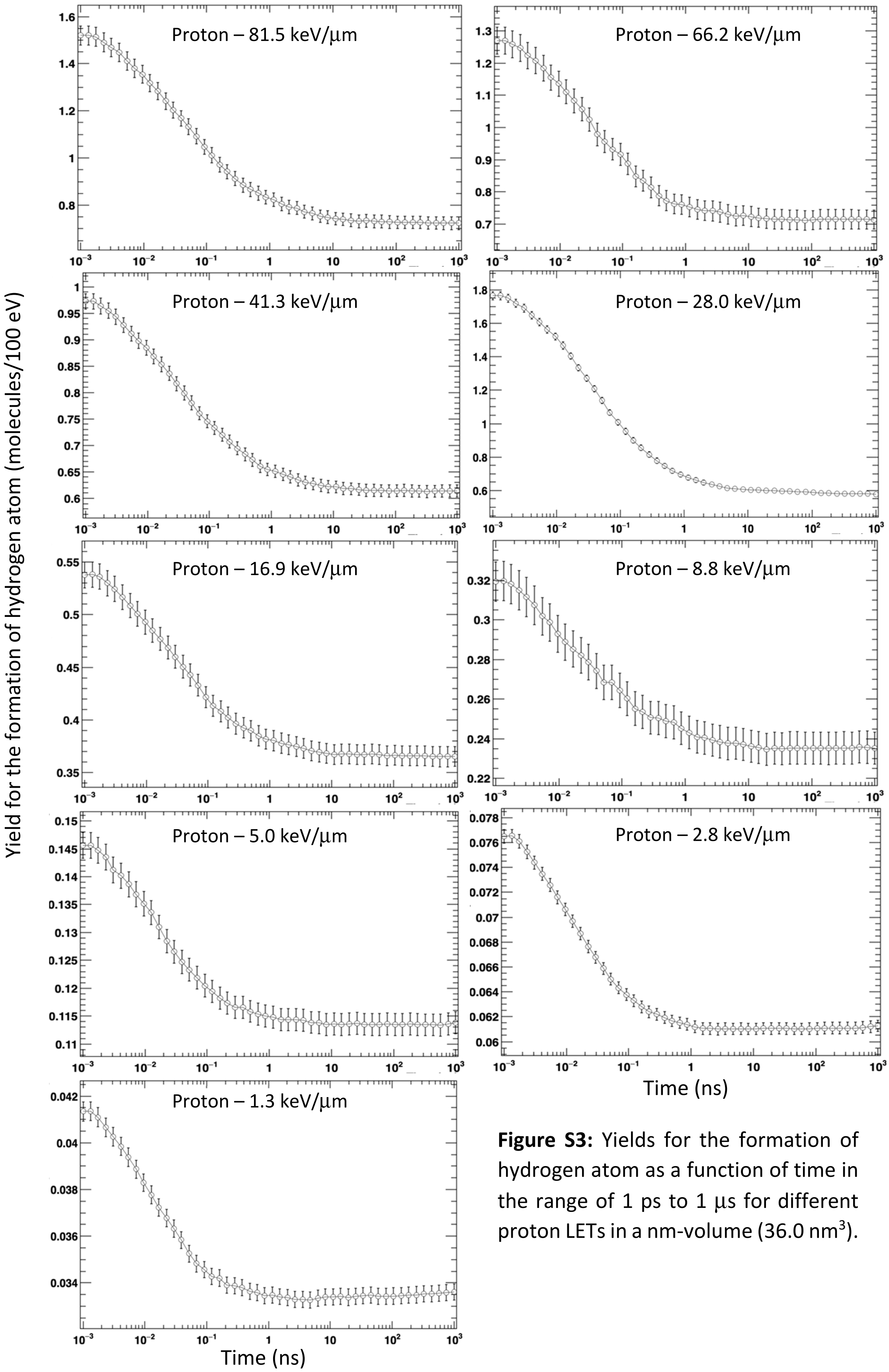

Figure S3: Yields for the formation of hydrogen atom as a function of time in the range of 1 ps to 1 μs for different proton LETs in a nm-volume (36.0 nm^3).

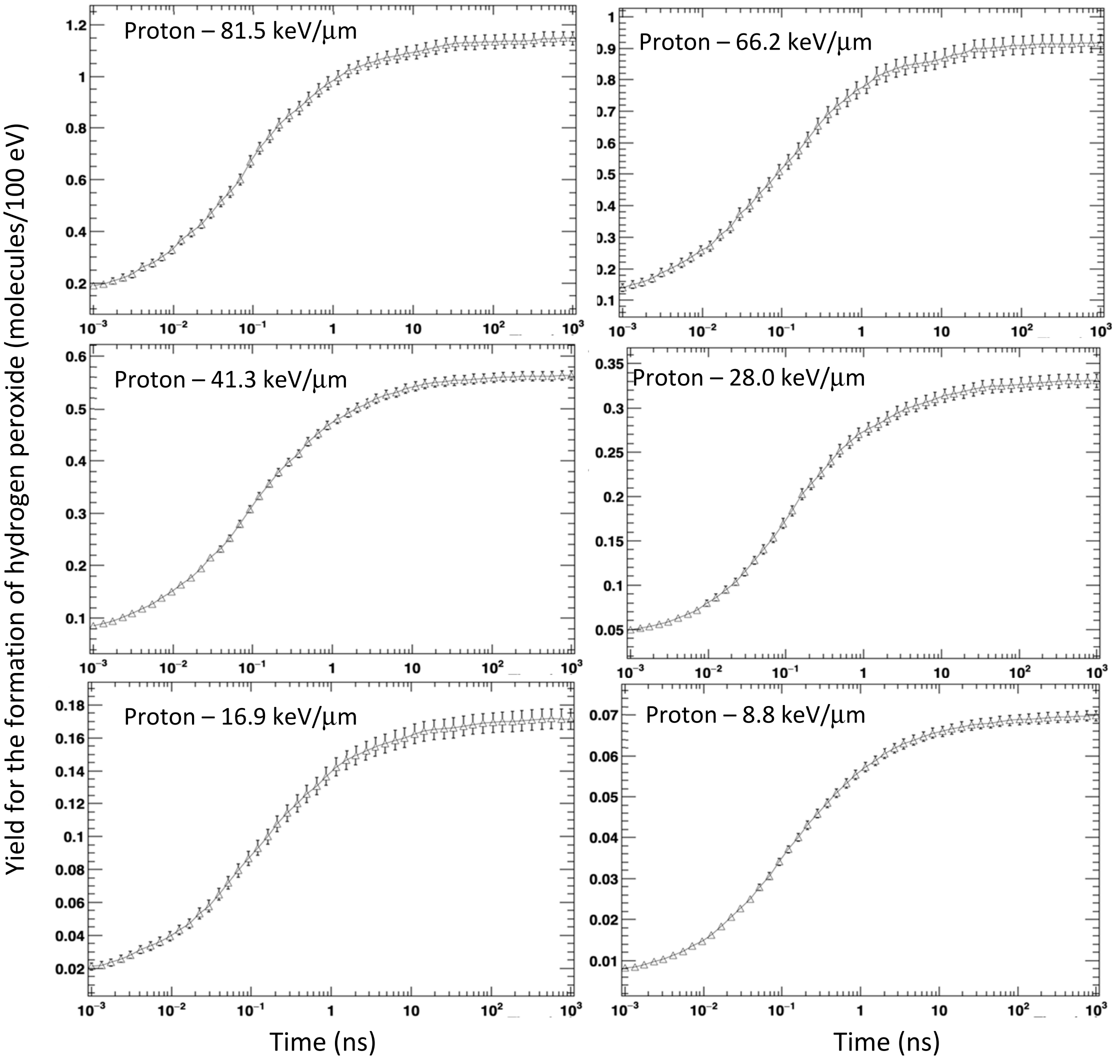

Figure S4: Yields for the formation of hydrogen peroxide as a function of time in the range of 1 ps to 1 μs for different proton LETs in a nm-volume (36.0 nm^3).

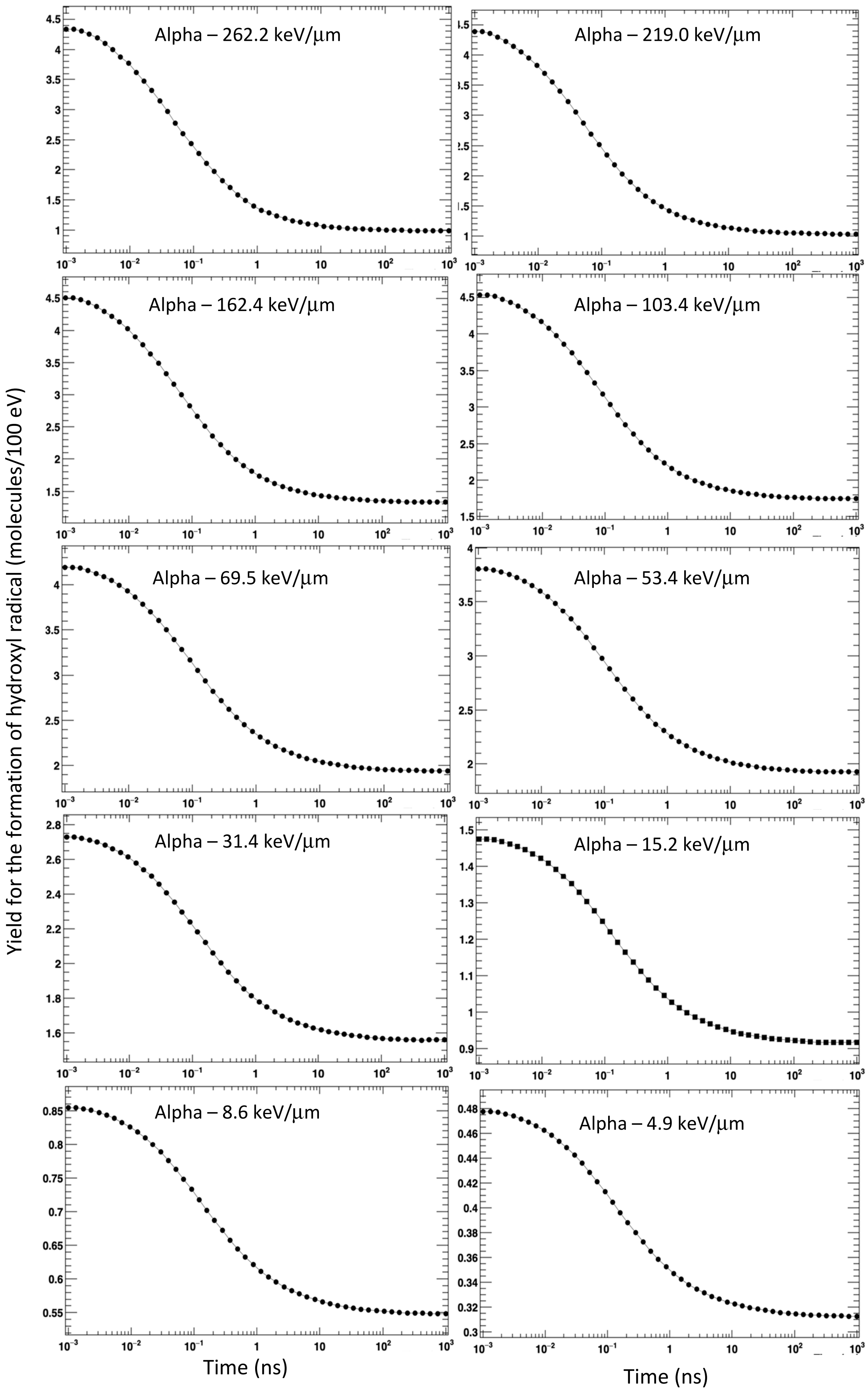

Figure S5: Yields for the formation of hydroxyl radicals as a function of time in the range of 1 ps to 1 µs by alpha particle at different LETs in a nm-volume (36.0 nm^3).

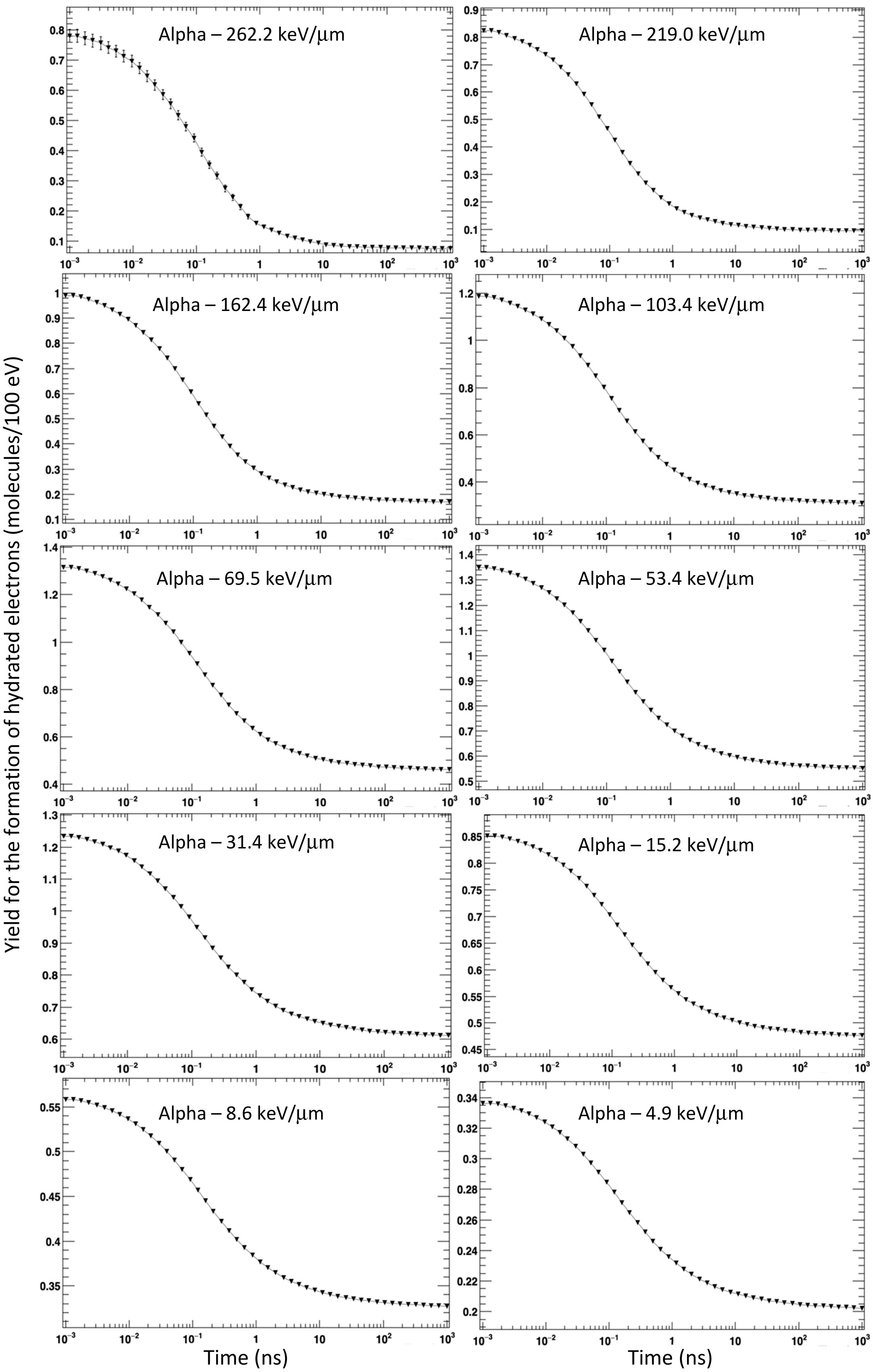

Figure S6: Yields for the formation of hydrated electrons as a function of time in the range of 1 ps to 1 μs by alpha particle at different LETs in a nm-volume (36.0 nm^3).

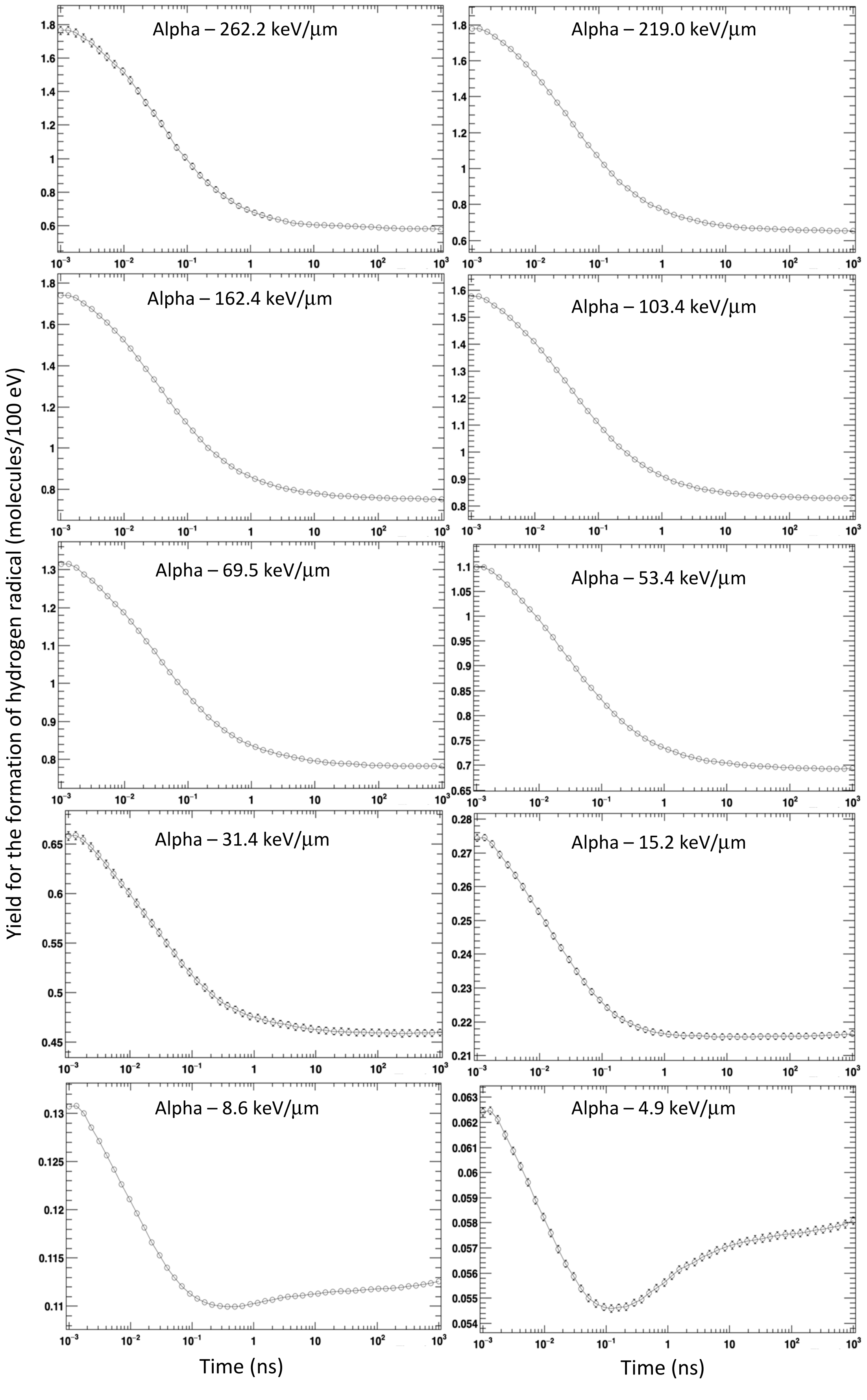

Figure S7: Yields for the formation of hydrogen radical as a function of time in the range of 1 ps to 1 μs by alpha particle at different LETs in a nm-volume (36.0 nm^3).

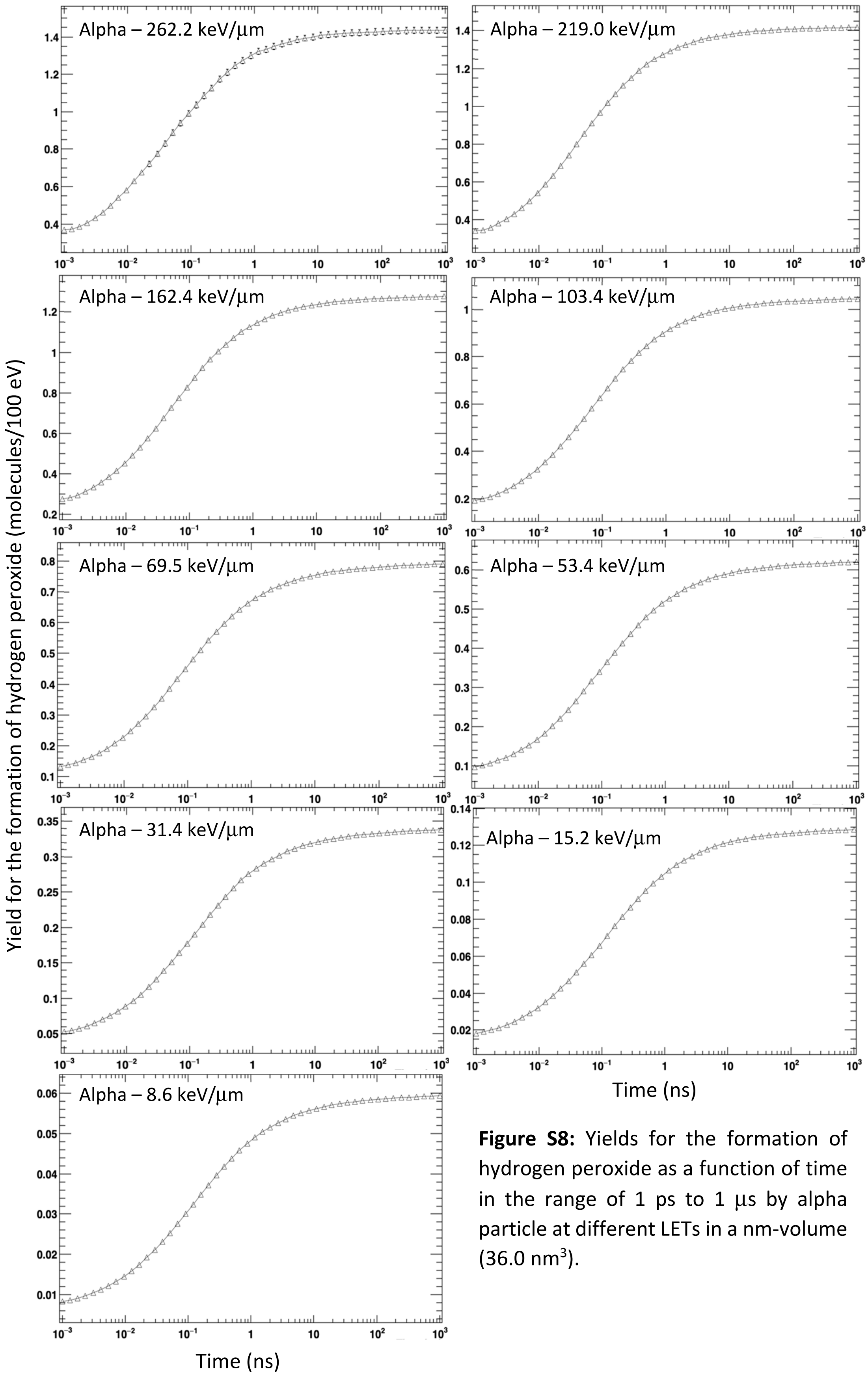

Figure S8: Yields for the formation of hydrogen peroxide as a function of time in the range of 1 ps to 1 μs by alpha particle at different LETs in a nm-volume (36.0 nm^3).
